# Imprinting and Promoter Usage of Insulin-Like Growth Factor II in Twin Discordant Placenta

**DOI:** 10.1155/2010/498574

**Published:** 2010-06-22

**Authors:** Yan-Min Luo, Qun Fang, Hui-Juan Shi, Lin-Huan Huang, Run-Cai Liang, Guang-Lun Zhuang

**Affiliations:** ^1^Fetal Medicine Center, Department of Obstetrics and Gynecology, First Affiliated Hospital of Sun Yat-sen University, Guangzhou, Guangdong 510080, China; ^2^Department of Pathology, First Affiliated Hospital of Sun Yat-sen University, Guangzhou, Guangdong 510080, China; ^3^Department of Obstetrics and Gynecology, First Affiliated Hospital of Sun Yat-sen University, Guangzhou, Guangdong 510080, China

## Abstract

Case reports from infant twins suggest that abnormal genomic imprinting may be one of the important causes of twin discordance, but it is unknown whether abnormal genomic imprinting occurs in the placenta. Therefore, we sought to determine the relationship between the imprinting of insulin-like growth factor II (IGF-II) in placenta and twin discordance. We analyzed the imprinting and promoter usage of IGF-II in placenta of normal twins (T0 group), weight discordance (T1 group), and phenotype discordance (T2 group). We found the incidence of loss of imprinting (LOI) for IGF-II was higher in the T2 group than that in the T0 and T1 groups, while there was no difference between T0 and T1 groups. The transcripts of promoter 3 were lower in the T2 group than in the T0 and T1 groups, and lower in the twin placenta with LOI than in those with normal imprinting. Our findings indicate that the promoter 3 specific LOI of the IGF-II gene may be closely related with phenotype discordance, not weight discordance.

## 1. Introduction

Twin discordance is a complication that is unique to multiple gestations. It is defined as discordance in the growth pattern, genotype, and phenotype of the twins. It can also just refer to a birth weight discordance of more than 20% between the twins [1]. Perinatal outcomes are worse in discordant twins than in concordant twins. Discordant twins have been found to have a significant increase in perinatal death, congenital anomalies, severe intracranial haemorrhage, neurological morbidity, neonatal asphyxia, and respiratory distress [2, 3]. It is important to study the causes of twin discordance in order to find a treatment and improve the prognosis. 

Abnormal genomic imprinting may be one of the important causes that results in twin discordance [4]. Genomic imprinting is a special phenomenon that operates outside of Mendel's law and directs the nonrandom monoallelic expression of specific autosomal genes according to their parental origin. Loss of imprinting (LOI) is an epigenetic alteration associated with the expression of the normally repressed parental allele or silence of the normally active allele. Imprinted genes play a key role in fetal and placental growth, development of particular lineages, and behaviour [5, 6]. It has been reported that an abnormal adjustment of genomic imprinting may result in congenital anomalies, some genetic diseases, and tumours [7, 8].

Insulin-like growth factor II (IGF-II) is one of the crucial imprinted genes related to fetal growth and placental development. It has 4 promoters and is expressed by the paternal allele. The expression of IGF-II is very complex since its imprinting is periodic, as well as tissue and promoter specific. Placental IGF-II can control fetal growth by modulating placental development and nutritional transfusion [9]. The LOI of the IGF-II gene is associated with fetal anomalies, while mutations of the IGF-II gene promoter result in fetal growth restriction [5].

Few studies have examined the changes of IGF-II in twin pregnancies, and there are no reports dealing with IGF-II imprinting and promoter regulation in twin placenta. It is uncertain whether IGF-II gene imprinting and promoter regulation in the placenta are associated with twin discordance. To clarify the role of imprinted IGF-II genes in the pathogenesis of twin discordance, the imprinting and promoter usage of IGF-II were studied.

## 2. Materials and Methods

### 2.1. Placenta Collection

Human placentas were collected after birth in the first Affiliated Hospital of Sun Yat-sen University (Guangzhou, China). Eighty sets of twins were enrolled, including 55 sets (64.71%) of normal twins (T0 group), 17 sets (20.00%) of twins with weight discordance (T1 group), and 13 sets (15.29%) of twins with phenotype discordance (T2 group). Weight discordance was defined as a birth weight discordance of more than 20% between the twins. Phenotype discordance was defined as being present when only one of the twins had a malformation or both twins had different abnormalities. Detailed description of T2 group was listed in Table 1.

For each twin, tissue samples were collected from under the umbilical cord even when the twins shared the same placenta. The tissues were washed with a saline solution to remove maternal blood, then frozen in liquid nitrogen, and stored at −80°C until analysis.

### 2.2. Preparation of Genomic DNA and RNA

Approximately 0.1 g of the frozen tissues was homogenized using Teflon tissue grinders. Genomic DNA (gDNA) was extracted using phenol and chloroform. Total RNA isolation was performed using TRI-reagent as suggested by the manufacturer (Molecular Research Center Inc, Cincinnati, OH, USA). The quality and quantity of the extracted RNA was assessed using an Ultraviolet Bioanalyzer (Eppendorf, Hamburg, Germany). Total RNA was treated with 10 Units of DNase I (Fermentas, Vilnius, Lithuania) for 30 minutes at 37°C to eliminate any genomic DNA residue.

### 2.3. Identification of Genomic Polymorphisms

To assess the heterozygosity of the IGF-II gene, Apa I polymorphism within exon 9 of IGF-II was screened. For PCR amplification of the IGF-II gene, the following primers were used: sense strand (P1F), 5′CTTGGACTTTGAGTCAAATTGG-3′, and antisense strand (P1R), 5′-CCTCCTTTGGTCTTACTGGG-3′. For each 50 *μ*l PCR, 2 *μ*l gDNA, 5 *μ*l 10×PCR buffer, and 0.5 U Taq DNA Polymerase (TaKaRa, Dalian, China), 1 *μ*l sense and antisense primer (10 pmol) and 1 *μ*l dNTPs (2.5 mM) were amplified using an initial denaturation step at 94°C for 7 minutes, followed by 35 cycles of denaturation at 94°C for 30 s, annealing at 60°C for 40 s with a 40 s extension, and a final extension at 72°C for 9 minutes. The amplification resulted in a gene fragment of 236 bp whose allele had an ApaI restriction site at 173 bp. The PCR products were digested with the restriction enzyme ApaI (New England Biolabs, Ipswich, UK) and loaded onto 3% agarose gel, which was followed by ethidium bromide staining.

### 2.4. Allele-Specific Expression of IGF-II Genes

Allele-specific expression of the IGF-II genes was examined using reverse transcription-PCR (RT-PCR) followed by enzyme digestion as described above. cDNA was synthesized using about 1 *μ*g RNA and reverse transcriptase reagents (RevertAid H Minus First Strand cDNA Synthesis Kit, Fermentas, Vilnius, Lithuania) as suggested by the manufacturer. The resulting cDNA samples were heated at 70°C for 10 minutes to inactivate the reverse transcriptase, and then used for PCR amplification as described above. The primers used for RT-PCR of IGF-II were P1F and P1R as described above. The PCR products were digested with ApaI (yielding either a 236 bp fragment or a 173 and 63 bp fragment), electrophoresed through a 3% agarose gel, and visualized with ethidium bromide.

### 2.5. Promoter-Specific Expressions of IGF-II

Promoter-specific expressions of IGF-II were quantified using the Absolute QPCR SYBR Green Mix plus ROX vial Kit (Abgene, Epsom, UK) and the OpticonTM 2 continuous fluorescence detection system (MJ Research, Boston, MA, USA). The primers used in real-time quantitative PCR (RT-PCR) for each promoter specific expression of IGF-II are listed in Table 2. The OpticonTM 2 was programmed as follows: preincubation and denaturation of template cDNA for 15 minutes at 94°C; followed by 40 cycles of amplification: 96°C for 15 s, 66°C for 40 s (P1) or 69°C for 20 s (P2) or 60°C for 30 s (P3) or 63°C for 30 s (P4), and 72°C for 40 s (P1) or 20 s (P2) or 30 s (P3, P4). The melting curve analysis was performed at 65°C to 98°C, 0.2°C /read, 2 seconds hold.

### 2.6. Statistics

The statistical analysis was performed using the *χ* ^2^ test, Fisher's exact test, Student's *t* *-*test, and ANOVA, as appropriate. A *P* value of less than  .05 was considered significant.

## 3. Results

### 3.1. Imprinting of IGF-II

**Figure 1:**
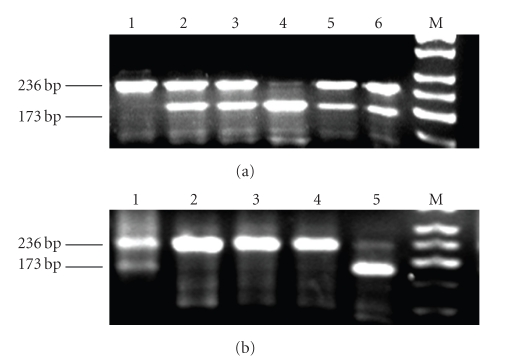
Polymorphisms and imprinting of the IGF-II gene in the placenta. (a) polymorphisms of IGF-II: 1 had type AA homozygosity; 4 had type BB homozygosity; 2,3,5,6 had type AB heterozygosity; and M, marker. (b) imprinting of IGF-II: 1, loss of imprinting AB; 2–4, normal imprinting A; 5, normal imprinting B; and M, marker.

For the imprinting study, DNA specimens from the placenta were first analyzed for heterozygosity using IGF-II gene polymorphisms at ApaI. Then, RT-PCR products were analyzed for LOI by biallelic expression of IGF-II ( Figure 1). The incidence of LOI of the IGF-II gene was 43.8% in the T2 group placenta, which was higher than that in the T0 and T1 groups (17.6% and 15.8%, resp., *P* < .05) (Table 3). All LOI occurred in abnormal twins except in one twin pair in which one twin was large for gestational age. 

There were 4 cases of twin-twin transfusion syndrome, including 1 acardiac twin. Among these 4 cases, 3 sets were IGF-II homozygous, and 1 set, which was a Stage III case of twin-twin transfusion syndrome, was IGF-II heterozygous. Polyhydramnios occurred in the recipient twin at 23 gestational weeks, and the maximal vertical amniotic fluid pocket was 16.5 cm. The donor twin, whose bladder was not visible, was a “stuck twin”, and the reverse end-diastolic velocity of the umbilical artery could be seen. The woman decided to abort the pregnancy. After induction, the imprinting status was analyzed. The donor twin had normal imprinting expression, while the recipient twin had LOI.

### 3.2. Promoter Usage of IGF-II

The usage of IGF-II promoters was similar in the T0, T1, and T2 groups. In each group, among the 4 promoters, the transcripts of IGF-II gene promoter 3 were the highest. The transcripts of IGF-II gene promoter 3 in group T2 were significantly lower than in the T0 and T1 groups (*P* < .05). The transcripts of IGF-II gene promoter 3 were lower in twin placenta with LOI than in those with normal imprinting (*P* < .05). Intrapair transcriptions of the 4 IGF-II gene promoters did not differ among the T0, T1, and T2 groups (*P* > .05). There was a positive correlation between the transcription of IGF-II gene promoter 3 and the transcription of IGF-II gene promoter 4 (*r* = 0.229, *P* < .05); there were no correlations with other promoters (*P* > .05).

## 4. Discussion

The paternally expressed, imprinted gene, IGF-II, located on chromosome 11 p15.5, encodes an autocrine growth factor that plays an important role in embryonic growth. LOI of the IGF-II gene results in generalized constitutional overgrowth, malformation, and a predisposition to the development of specific embryonal tumours, most commonly Wilm's tumour. Our data shows that the incidence of LOI of the IGF-II gene was higher in T2 than in T0 and T1 group placenta, and that all LOI occurred in abnormal twins, except in one case. The incidence of LOI of the IGF-II gene was similar in the T0 and T1 groups. Ravenel et al. [10], observed that there was virtually complete segregation of the intralobar nephrogenic rest (ILNR)-like and perilobar nephrogenic rest (PLNR)-like tumours, depending on the imprinting status; 9 (90%) of 10 PLNR-like tumours had LOI, but only 1 (6.7%) of 15 ILNR-like tumours had LOI, although the ILNR-like and PLNR-like tumours were both Wilm's tumours. Thus, it is possible that the pathology involved in phenotype discordant twins and weight discordant twins is different, and that LOI of the IGF-II gene may be closely related with phenotype discordant twins, as in PLNR-like tumours. Among the weight discordant twins, most of the small fetuses were selective fetal growth restriction and the larger ones were appropriate for gestational age, not overgrowth. Furthermore, LOI of the IGF-II gene is much more relevant to classical cases of Beckwith-Wiedemann syndrome (BWS) or Silver-Russell syndrome (SRS) than to sporadic growth deficiencies or overgrowth. These might be the possible reason why LOI of the IGF-II gene in placentae is not a major contributor to weight discordance.

Placental IGF-II regulates the growth and transport capacity of the placenta, thereby controlling the supply of nutrients. It may also directly regulate the growth rate of fetal tissues, thereby controlling fetal nutrient demand. LOI of the IGF-II gene in the placenta might alter the balance between placental and fetal growth and lead to fetal abnormalities. It is reported that LOI of the IGF-II gene was relevant to malformation like macroglossia and exomphalos, but not other malformation. The malformation cases in our data are diverse. It is not known whether fetal abnormalities are due to a direct effect of LOI of the IGF-II gene in the placenta, or whether LOI of the IGF-II gene in the placenta reflects LOI of the IGF-II gene in other fetal tissue, even other gene imprinting. Further investigation of methylation of other related imprinting genes is warranted to delineate the possible mechanism of the diverse malformation. And a larger series of phenotype discordant twins should be followed up, in which each type of specific malformation is represented by more cases.

During the life cycle of the organism, genomic imprints change in characteristic ways. They undergo erasure, establishment, and maintenance of methylation imprints at imprinting centers during germ cell and embryonic development. Imprint patterns are maintained as chromosomes duplicate and segregate, although there is genome-wide demethylation after fertilization and a wave of de novo methylation after implantation. Any changes in the microenvironment around the implantation could interfere with the mechanism of maintenance, so twins might be more sensitive to get LOI with the epigenetic risks related to assisted reproductive technologies and monochorionic twins. As the changes occur around the implantation, imprints may sometimes be discordant in twins, even in monozygotic twins. 

In our data, among 4 cases of twin-twin transfusion syndrome, 3 sets were homozygous for IGF-II, and 1 set was heterozygous (the donor twin had normal imprinting expression, while the recipient twin had LOI). There have been no reports dealing with the relationship between twin-twin transfusion syndrome and LOI. Bajoria et al. [11], found that fetal IGF-II levels in recipient twins with TTTS were higher than those in donor twins. It was also reported that IGFs may be involved in endothelial dysfunction [12]. Thus, more research is needed to determine whether LOI of the IGF-II gene induced the abnormality of the vessels in the twin placenta that leads to twin-twin transfusion syndrome or was just related to the complications, such as twin-twin transfusion syndrome with abnormal vessels. 

IGF-II is transcribed from four distinct promoters, P1–P4. It has been found that the fetal liver uses only three promoters (P2, P3, and P4) for IGF-II transcription, with the P3 promoter having the highest activity [13, 14]. Late placental tissues of twin pregnancies show similar use of the IGF-II promoter as in fetal liver. The P3 transcript levels were higher than those of P4, while the P2 and P1 transcript levels were low or zero. Compared to normal twins, the P3 transcript levels in the T2 group were dramatically lower. This suggests that P3 plays the most important role in the placenta, and that its abnormal activity may affect fetal growth.

Usually, P1 has biallelic expression, and P2-4 have paternally monoallelic expression [15, 16]. It has been proposed that IGF-II expression through promoter P1 could explain the biallelic expression patterns in some neoplasms [17, 18]. In these neoplasms, P1 plays the most important role, and most transcripts are from P1 rather than P2-4. However, we found that, when there was LOI of the IGF-II gene in twin placenta, the activity of P3, not P1, was greatly changed. This suggests that methylation changes of P3 induce biallelic expression and decrease P3 activity. The mechanism of LOI of the IGF-II gene in phenotype discordant twins may be different from that in some neoplasms. For phenotype discordant twins, it was the biallelic expression of P3 and not P1 that caused the discordance. 

In conclusion, our data suggests that the promoter 3 specific LOI of the IGF-II gene may be closely related with phenotype discordance, not weight discordance. Further investigation of methylation of other related imprinting genes and more phenotype discordant twins was warranted to delineate the possible mechanism of the diverse malformation. Following up the weight discordance twins with LOI will find out whether they are predisposed to embryonal tumours.

## Figures and Tables

**Table 1:** Detail description of phenotype discordant in T2 group.

No.	Chorion	Twin A			Twin B		
		Abnormity	PM*	I**	Abnormity	PM*	I**
1	MCMA	Transposition of the great vessels, ventricular septal defect	AB	AB	N	AB	AB
2	DCDA	Mass on right back	AB	AB	N	AB	B
3	DCDA	Hydatidiform mole	AB	AB	N	AB	A
4	DCDA	Intrauterine death	AB	AB	N	AB	B
5	DCDA	Anencephaly, spinal bifida, omphalocele	AB	AB	N	BB	
6	MCDA	Thoracogastroschisis, ventricular septal defect, aortic overriding	AB	AB	N	AB	B
7	DCDA	Fetal hydrops	AB	A	N	AA	
8	DCDA	Hypospadias	AB	A	N	AA	
9	DCDA	Abdominal-wall defect, spinal bifida	AB	A	N	BB	
10	DCDA	Double outlet right ventricle	AB	B	N	AB	B
11	DCDA	Omphalocele	AA		N	AA	
12	MCDA	Acardiac twin	AA		N	AA	
13	MCDA	Hypospadias	AA		N	AA	

*AA, BB, and AB are three different gene polymorphisms; AA/BB reflects homozygosity, and AB reflects heterozygosity. **A, B, and AB are three different imprinting states; A/B is the normal imprinting expression, while AB reflects loss of imprinting. Abbreviations: Polymorphisim (PM), Imprinting (I), monochorionic monoamniotic twin (MCMA), dichorionic diamniotic twin (DCDA), monochorionic diamniotic twin (MCDA), and normal (N).

**Table 2:** Primers for promoter-specific expression of the IGF-II gene and internal control.

Name	Sequence (5′-3′)	Length
P1 (F)	AG TCC TGA GGT GAG CTG CTG	181 bp
P2 (F)	ACC GGG CAT TGC CCC CAG TC	277 bp
P3 (F)	ACA TTC GGC CCC CGC GAC TC	201 bp
P4 (F)	TCC TCC TCC TCC TGC CCC AG	118 bp
P1-4(R)	CAA TGC AGC ACG AGG CGA AG	
B-actin(F)	GCG GGA AAT CGT GCG TGA CAT T	228 bp
B-actin(R)	GAT GGA GTT GAA GGT AGT TTG GTG	

**Table 3:** Polymorphisms and imprinting of the IGF-II gene in placenta [n (%)].

Group	Polymorphisim			Imprinting		
	*n*	AA/BB*	AB*	*n*	A/B**	AB**
T0 group	110	41 (37.3)	69 (62.7)	68	56 (82.4)	12 (17.6)
T1 group	34	15 (44.1)	19 (55.9)	19	16 (84.2)	3 (15.8)
T2 group	26	10 (38.5)	16 (61.5)	16	9 (56.2)	7 (43.8)^#^

*AA, BB, and AB are three different gene polymorphisms; AA/BB reflects homozygosity, and AB reflects heterozygosity. **A, B, and AB are three different imprinting states; A/B is the normal imprinting expression, while AB reflects loss of imprinting. In the T0 group, the imprinting status could not be detected in one case with AB gene polymorphism.^#^ The frequency of loss of imprinting of the IGF-II gene was statistically significantly different between the T0 group and the T2 group, *P* = .035.
